# Amniotic Fluid Extracellular Vesicle Properties Evolve With Gestational Age and Reflect Fetal Development

**DOI:** 10.1002/jex2.70085

**Published:** 2025-10-08

**Authors:** Ishara Atukorala, Sally Beard, Ching‐Seng Ang, Hamish Brown, Swetha Raghavan, Natasha de Alwis, Bianca Fato, Natalie Binder, Natalie Hannan, Lisa Hui

**Affiliations:** ^1^ Department of Obstetrics, Gynaecology and Newborn Health, Melbourne Medical School The University of Melbourne Heidelberg Victoria Australia; ^2^ Mercy Perinatal Mercy Hospital for Women Heidelberg Victoria Australia; ^3^ Northern Health Epping Victoria Australia; ^4^ Walter and Eliza Hall Institute of Medical Research Parkville Victoria Australia; ^5^ Bio21 Molecular Science and Biotechnology Institute The University of Melbourne Parkville Victoria Australia; ^6^ Therapeutics Discovery and Vascular Function in Pregnancy Group, Department of Obstetrics, Gynaecology and Newborn Health, Melbourne Medical School The University of Melbourne Heidelberg Victoria Australia; ^7^ Murdoch Children's Research Institute Parkville Victoria Australia

**Keywords:** extracellular vesicles, amniotic fluid, fetal physiology, pregnancy, proteomics

## Abstract

Amniotic fluid (AF) is a valuable source of extracellular vesicles (EVs) derived from the fetoplacental unit. Preclinical and clinical studies have highlighted promising applications of AF‐EVs and their role in cellular communication, yet our understanding of AF‐EV physiology remains limited. This study aimed to examine the physiological importance of AF‐EVs in fetal development from the second trimester to term gestation. We obtained AF samples from routine second‐trimester amniocentesis and prelabour Caesarean section at term. We isolated EVs using a combination of differential centrifugation, filtration and ultracentrifugation and characterised them using nanoparticle tracking analysis, cryo‐electron microscopy and Western blotting. The differential EV proteome was analysed using label‐free proteomics. We assessed the second trimester and term AF‐EV properties through an enrichment analysis. The EV size and protein enrichment difference revealed a gestational‐age‐dependent variation in the predominant EV subtype. Second‐trimester‐derived EVs were enriched in ectosomes, while term EVs contained a significant proportion of exosomes. We identified several morphologies of AF‐EVs, including unilamellar, multilamellar, multicompartmental and granular‐centred EVs, across gestational ages. Proteomics analysis of AF‐EVs identified 4137 proteins with high confidence, of which 1090 exhibited significant differential enrichment between the two groups. Second‐trimester‐enriched AF‐EV proteins represented molecule assembly processes, metabolism and organogenesis. At term, AF‐EV proteins corresponded to impending newborn functions such as immunity and digestion. In conclusion, we provide compelling evidence that EV biogenesis and secretion in the fetoplacental unit undergo significant alterations across gestational ages, revealing a complex and dynamic physiology and intercellular communication that adapts to the needs of the developing fetus.

## Graphical abstract

This figure summarises the study's main findings, including the shift in the predominant EV subtype, the different organs and biofluids represented by the enriched proteins at each gestational age, and the main biological pathways.

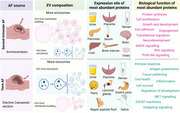

## 1 Background

Extracellular vesicles (EVs) are released from all living cells as a natural aspect of their cellular homeostasis. They are membrane‐enclosed vesicles that contain selectively packed cargo of biologically active molecules such as proteins, nucleic acids and lipids (Colombo et al. 2013, Atukorala and Mathivanan 2021). EVs mediate intercellular communication via this internal cargo and membrane‐bound molecules. Their role in facilitating inter‐organ communication, coordinating physiological processes and maintaining systemic homeostasis is widely studied (O'Brien et al. 2020, Valadi et al. 2007, Radnaa et al. 2021). EV biogenesis pathways influence cargo sorting (Raposo et al. 1996) and change in response to extracellular stimuli (Hazelton et al. 2018, Bei et al. 2017). However, the mechanisms driving the change in EV cargo composition, particularly in vivo, are poorly understood (Yates et al. 2022).

Amniotic fluid (AF) is a valuable sample with a wealth of fetal biological information (Underwood et al. 2005, Moore 2010, Hui et al. 2012). In early gestation, AF primarily consists of maternal plasma ultrafiltrate. By 8–10 weeks, fetal urine production and swallowing begin, shifting AF composition towards greater fetal contribution (Liu et al. 2008, Ross et al. 2023). AF constantly circulates the fetal body by urination, lung fluid secretion and swallowing (Ross and Nijland 1997). AF volume increases until about 32 weeks, then gradually decreases. In the near term, the fetus produces 700–1000 mL of urine daily, with complete fluid turnover occurring every 24 h (Bianchi et al. 2010, Tong et al. 2009). AF also has immune properties and reflects fetal immune status, offering diagnostic potential in infection‐related pregnancy complications. It contains essential nutrients and growth factors, including electrolytes, proteins and amino acids, that support growth, regulate development and maintain metabolic balance (Underwood et al. 2005, Atukorala et al. 2024). Collectively, AF plays a vital role in fetal nutrition, development and homeostasis.

AF is used clinically for prenatal diagnosis, primarily for amniocyte analysis for chromosome and genetic abnormalities (Anandakumar et al. 1992, Wang et al. 2018). However, AF‐EVs have been relatively unexplored until recently. Numerous exploratory studies have been published in the field of AF‐EV research (Atukorala et al. 2024), showing substantial potential for translational impact in prognosis and treatment in perinatal medicine. The lipid bilayer membrane of EVs protects their cargo from external degrading enzymes (Colombo et al. 2014), suggesting that EVs can contain stable biomarkers for understanding health and disease (Gebara et al. 2022, Dixon et al. 2018). A growing body of preclinical and clinical researchers is now investigating the anti‐inflammatory, antioxidative, regenerative and angiogenic molecules within EVs for therapeutic applications (Cargnoni et al. 2021, Bellio et al. 2021, Senesi et al. 2024, del Rivero et al. 2022, Nunzi et al. 2023). Conversely, our understanding of the general physiology of EVs from the fetoplacental unit is limited, including the cells they represent, the molecular information they contain, and the likely purpose of this information for both the baby and the mother.

This study aimed to delineate and compare AF‐EVs derived from uncomplicated second‐trimester and term pregnancies to uncover their physiology. These findings may enhance our understanding of AF‐EV biogenesis, cargo and physiological significance in fetal growth and development.

## 2 Methods

### 2.1 Amniotic Fluid Sample Collection

AF samples were collected prospectively at Mercy Hospital for Women in Melbourne, Australia, following written informed participant consent. Second‐trimester samples were collected from clinically indicated amniocentesis due to abnormal prenatal screening or sonography results (Table 1). Term samples were collected from pre‐labour Caesarean sections of uncomplicated pregnancies, after the uterine incision prior to membrane rupture, as previously described (Hui et al. 2013). All samples were free from contamination, including maternal blood and meconium. The AF samples were centrifuged at 300 × *g* for 10 min to remove cells and debris before being stored at −80°C until EV isolation. This study was approved by the Human Research Ethics Committee of Mercy Health (2023‐014).

**TABLE 1:** Clinical characteristics of the samples.

Second‐trimester samples			
Gestational age at collection (weeks)	Fetal sex	Indication for amniocentesis/Caesarean section	Maternal age at collection (years)
15.4	F	Bilateral cleft lip and palate	34
15.4	M	Thin nuchal translucency (4.5 mm)	36
16.1	M	Increased chance of trisomy 21 detected in non‐invasive prenatal testing	34
16.2^a^	F	Increased chance of trisomy 21 detected in first‐trimester combined screening	28
23.0	M	Bilateral mild cerebral ventriculomegaly	34
23.1	F	Increased chance of trisomy 21 detected in maternal serum screening	35
Full‐term samples			
38.2	M	Previous Caesarean section	29
39.0	F	Previous Caesarean section	39
39.0	F	Previous Caesarean section	30
39.3	M	Previous Caesarean section	31
39.0	M	Previous Caesarean section	36
39.3	F	Previous Caesarean section	29

*Note*: F, female; M, male.

^a^Birth via Caesarean section at 36 weeks of gestation due to placenta praevia. Other participants in the second‐trimester amniocentesis group delivered healthy live newborns at term gestation.

### 2.2 EV Isolation

AF‐EVs were isolated using differential centrifugation, filtration and ultracentrifugation. EVs were characterised according to MISEV23 guidelines (Welsh et al. 2024).

AF samples were thawed overnight at 4°C and centrifuged at 5000 × *g* for 20 min at 4°C to remove vernix caseosa and other debris. Supernatant was centrifuged at 15,000 × *g* for 30 min at 4°C, then filtered through a 0.22‐µm filter. The resulting supernatant was subjected to ultracentrifugation at 120,000 × *g* for 2 h at 4°C to pellet small EVs. EV pellet was then washed with 0.22 µm‐filtered Dulbecco's phosphate‐buffered saline (DPBS; Gibco) and centrifuged at 120,000 × *g* for 2 h at 4°C. The resulting EV pellet was resuspended in filtered DPBS and stored at −80°C until further analysis.

### 2.3 EV Protein Quantification

EV proteins (2 µg) of each sample immobilised on a polyacrylamide gel were fixed with fixer buffer (50% methanol, 7% acetic acid) and stained with Sypro Ruby gel stain (Thermo Fisher Scientific) for 24 h. After washing the gel with wash buffer (10% methanol, 7% acetic acid), the gel was scanned using Chemidoc (Bio‐Rad) and the lanes were quantified against the Benchmark protein ladder (Thermo Fisher Scientific).

### 2.4 Nanoparticle Tracking Analysis

EV size range and concentration were determined using NanoSight NS300 (Malvern Panalytical; NanoSight NTA 3.2 software). Samples were diluted 400‐fold with 0.22 µm‐filtered PBS and injected at an infusion rate of 50. Each sample was captured in 3 rounds of 30 s, with camera level and detection threshold set at 11 and 5, respectively, at 25°C.

### 2.5 Western Blotting

EVs corresponding to 15 µg of protein were lysed in 4x Laemmli buffer (8% (w/v) SDS, 10% (v/v) glycerol, 200 mM Tris‐HCL, pH 6.8 and a trace of bromophenol blue) and 2 M DTT, heated at 95°C for 2 min and separated on 4%–15% polyacrylamide gels (Bio‐Rad). Gels were electrophoresed at 120 V for ∼90 min. Proteins were transferred to PVDF membranes (Thermo Fisher Scientific) using a wet electroblotting system (Bio‐Rad), 100 V, 1 h and blocked with 5% (w/v) skim milk in Tris‐buffered saline with Tween 20 (TBST) for 1 h. After washing the membranes 3 times, 10 min each in TTBS, they were incubated with primary antibodies for Alix (Catalogue # E6P9B, 1:1000, Cell Signalling), CD9 (Catalogue # 10626D, 1:500, Thermo Fisher Scientific) and CD63 (Catalogue # 10628D, 1:500, Thermo Fisher Scientific) at 4°C overnight. After washing the membranes 3 times, 10 min each in TBST, they were incubated with the relevant fluorescent‐conjugated secondary antibodies (IRDye 680RD Goat anti‐Rabbit IgG or IRDye 800CW Goat anti‐Mouse IgG) (LICORbio) in a 1:10,000 dilution for 1 h at room temperature. Following washing the membranes three times, 10 min each in TBST, they were imaged using ChemiDoc (Bio‐Rad).

### 2.6 Cryo‐Electron Microscopy and Image Analysis

Cryo‐electron microscopy was used to visualise vesicles. Gold 300 mesh, lacey carbon film‐coated EM grids (ProSciTech, Australia) were glow‐discharged (15 mA, 30 s) using the GloQube Plus Glow Discharge System. A sample (3 µL of initial EV preparation) was applied onto the carbon side of the grid, which was then blotted for 4.0 s (−1 bolt force) with Whatman filter paper #1 and plunge‐frozen into liquid ethane using a Thermo Fisher Vitrobot Mark IV. The climate chamber was maintained at 90% humidity, 4°C. EM grids containing frozen samples were stored in liquid nitrogen until imaging. EVs were visualised using a Thermo Fisher TECNAI F30 cryo‐electron microscope, equipped with a Ceta electron camera, at the Ian Holmes Imaging Centre (The University of Melbourne). Images were recorded at x2400 magnification with a defocus of −5 µm and 10 Ȧ pixel size.

Image analysis tool Fiji ImageJ (Schindelin et al. 2012) was used to measure the diameter of EVs. First, the scale bar of each image was defined by the number of pixels under the ’set scale’ function. The average of the major (largest) and minor (shortest) EV axes was used as the measure of EV size, in keeping with common practice in the field (Emelyanov et al. 2020, Noble et al. 2020).

### 2.7 Sample Preparation for Mass Spectrometry

EV proteins (30 µg) were lysed in 2X lysis buffer (10% SDS, 100 mM TEAB, pH 8.5). Disulphide bonds of the proteins were reduced using tris‐(2‐carboxyethyl)phosphine (final concentration 5 mM) (Thermo Fisher Scientific) and alkylated using Methyl methanethiosulfonate (final concentration 20 mM). Samples were acidified with a final concentration of ∼2.5% phosphoric acid. The samples were loaded onto S‐Trap micro spin columns (ProtiFi, USA) with binding/wash buffer (100 mM triethylammonium bicarbonate in 90% methanol) and centrifuged at 4000 × *g* for 30 s to trap proteins. After washing the columns 3 times with 150 µL binding/wash buffer, proteins were digested with Sequencing Grade Modified Trypsin (Promega) at a ratio of 1:10 (w/w) trypsin:protein for 2 h at 47°C. Peptides were collected consecutively in 3 elution steps with 50 mM TEAB in water, 0.2% formic acid in water and 50% acetonitrile in water. Eluted peptides were lyophilised using a vacuum concentrator (Savant SpeedVac) and reconstituted in mass spectrometry sample buffer (2% acetonitrile, 0.05% trifluoroacetic acid) to a final peptide concentration of 0.5 µg/µL.

### 2.8 Data‐Independent Acquisition (DIA) Mass Spectrometry (MS)

Liquid chromatography (LC)‐based tandem mass spectrometry (MS/MS) was carried out using an Orbitrap Ascend mass spectrometer (Thermo Fisher Scientific) equipped with a nanoflow reversed‐phase HPLC (Ultimate 3000 RSLC, Dionex), fitted with an Acclaim Pepmap nano‐trap column (Dionex—C18, 100 Å, 75 µm × 2 cm) and an Acclaim Pepmap RSLC analytical column (Dionex‐C18, 100 Å, 75 µm × 50 cm), at the Melbourne Mass Spectrometry and Proteomics Facility (The University of Melbourne). The tryptic peptides (0.5 µg) were injected into the enrichment column at an isocratic flow of 5 µL/min of 2% v/v CH_3_CN containing 0.1% v/v formic acid for 5 min before the enrichment column was switched in line with the analytical column. The eluents for LC were 5% DMSO in 0.1% v/v formic acid (solvent A) and 5% DMSO in 100% v/v CH_3_CN and 0.1% v/v formic acid (solvent B). The flow gradient was (i) 3% B for 0–6 min, (ii) 3%–4% B for 6–7 min, (ii) 4%–25% B for 7–82 min, (iii) 25%–40% B for 82–86 min, (iv) 40%–80% B for 86–87 min, (v) 80%–80% B for 87–90 min and (vi) 80%–3% for 90–91 min. The column was equilibrated at 3% B for 10 min before the next sample injection.

For DIA experiments, full MS resolutions were set to 120,000 at m/z 200 and scanning from 350–1400 m/z in the profile mode. Full MS Automatic Gain Control target was 250% with an injection time (IT) of 50 ms. AGC target value for fragment spectra was set at 2000%. Fifty windows of 13.7 Da were used with an overlap of 1 Da. Resolution was set to 30,000 and maximum IT to 55 ms. The normalised collision energy was set at 30%. All data were acquired in centroid mode using positive polarity.

### 2.9 Proteomics Data Analysis

DIA data were analysed using the direct DIA analysis workflow with default settings on the Spectronaut software (v. 17.5.230413.55965) and against the UniProt Homo Sapiens database (updated September 2023). Trypsin specificity was set to two missed cleavages. Carbamidomethyl (Cys) was defined as the fixed modification, while acetylation (protein N‐term) and oxidation (Met) were defined as variable modifications. Results were filtered at a protein and peptide level, and spectra were matched with a false discovery rate of 1%. Precursor filtering used the Q value, and quantification was done at the MS2 level. The cross‐run normalisation strategy was set to automatic. Pre‐ and post‐normalisation plots are provided in Figure S1. Quantitative data were exported (Spectronaut output at 1% FDR is available as Supporting Information, File 1) for statistical analyses onto MaxQuant Perseus (Max‐Planck‐Institute of Biochemistry) (Tyanova and Perseus 2018). The data were log2‐transformed and annotated into two groups: second trimester and full‐term. The rows were filtered to include the proteins identified in all 12 samples. Ubiquitously detected peptides in all samples were subjected to the Student's *t*‐test (with a false discovery rate of 0.05 and S_0_ filter set to 0.1) to determine significantly differentially abundant proteins between groups.

For the proteins uniquely detected at the two gestational time points, a PubMed search was conducted to identify their role in fetal growth and development. FunRich version 3.1.4 was used to perform the enrichment analysis. It is a functional enrichment analysis tool that integrates multiple databases, including UniProt, Human Protein Reference Database (HPRD) and Human Protein Atlas (Pathan et al. 2017). To keep the interpretation relevant to our fetal samples (Hui et al. 2013), enrichment results specific to adult pathologies and transformed cell lines were excluded.

All experimental details are available on the EV‐TRACK database (Van Deun et al. 2017) with the identifier EV250076.

### 2.10 Statistical Analysis

The statistical analysis for all experiments except proteomics was conducted using GraphPad Prism 10.1.0 (GraphPad Software). We determined the normal distribution of the data using the Shapiro–Wilk test. Unpaired two‐tailed Student's *t*‐test and the Mann–Whitney *U* test were used for parametric and nonparametric datasets, respectively.

## 3 Results

### 3.1 Clinical Characteristics of the Samples

Twelve AF samples were analysed: six from second‐trimester amniocenteses and six from term prelabour Caesarean sections. Each group comprised three female and three male fetuses. Of the second‐trimester group, three underwent amniocentesis for an increased chance of trisomy 21 on prenatal screening and others due to fetal ultrasound anomalies. One 23‐week male fetus with mild bilateral ventriculomegaly had no additional findings on fetal brain magnetic resonance imaging (MRI), resolution of the ventriculomegaly by the third trimester, and no abnormalities on newborn cranial ultrasound. One fetus had an isolated increased nuchal translucency measurement of 4.5 mm that resolved by the second trimester. The third fetus with an ultrasound abnormality had an isolated cleft lip and palate. All fetuses in the amniocentesis group had normal molecular karyotypes. The full‐term group underwent elective Caesarean sections due to their history of previous Caesarean sections (Table 1).

### 3.2 Amniotic Fluid EVs in the Second Trimester and Term Differ in Size and Biogenesis

**FIGURE 1:**
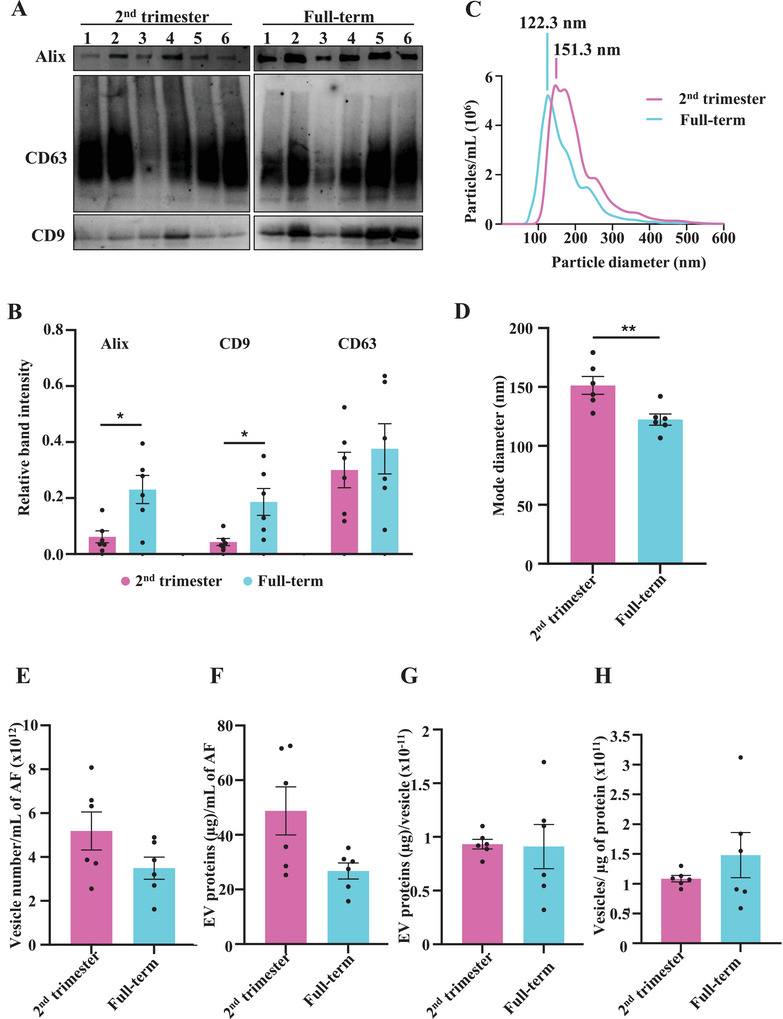
Amniotic fluid EVs at term are smaller in diameter compared to second trimester. (A) AF‐EVs were characterised using Western blotting for EV‐enriched markers Alix, CD9 and CD63. (B) Densitometric analysis of the Western blot confirmed the enrichment of Alix and CD9 in terms AF‐EVs. (C) Size distribution of AF‐EVs was analysed by nanoparticle tracking analysis. The graphs indicate (D) the mode diameter of the AF‐EVs, (E) AF‐EV concentration, (F) EV protein concentration in AF, (G) EV protein amount (µg) per vesicle and (H) Particles/µg of protein. Statistical analysis used the Student's *t*‐test with a confidence interval of 95%, in GraphPad Prism 10.1.0 (316). Error bars represent mean ± SEM. *<0.05 **<0.01. AF‐EVs, amniotic fluid‐extracellular vesicles.

Western blotting confirmed the presence of EV‐enriched protein markers Alix, CD9 and CD63 in the isolated EV samples (Figure 1A). Alix is a protein associated with the EV biogenesis pathway (ESCRT), while CD9 and CD63 are tetraspanins (Welsh et al. 2024, Mathieu et al. 2021). The relative quantities of Alix and CD9 (normalised to the total blot stain in Figure S2A) were significantly higher in the term AF‐EVs (Figure 1B). Complete blots for Alix, CD63 and CD9 are in Figure S2B,C. The enrichment of Alix suggests a greater fraction of endosomal origin EVs (exosomes) in term AF‐EVs compared to that of the second trimester.

The mode diameter identified by nanoparticle tracking analysis for the second trimester (151.3 nm) was significantly higher compared to that of term AF‐EVs (122.3 nm) (Figure 1C,D). The EV concentration of AF stayed unchanged between the two gestation time points (Figure 1E). Quantification of EV proteins revealed that neither the EV protein concentration in AF (Figure 1F) nor the protein amount (µg) per vesicle (Figure 1G) changed according to gestational age. We confirmed the purity of the EV preparations by analysing the particle‐to‐protein ratio (Figure 1H). The ratios were in the range of 5.89 × 10^10^ to 3.12 × 10^11^ particles/µg, above the accepted threshold for high vesicular purity (Webber and Clayton 2013). Also, there was no significant difference in the level of purity between the two groups.

### 3.3 Amniotic Fluid EVs Are Highly Heterogeneous in Morphology

**FIGURE 2:**
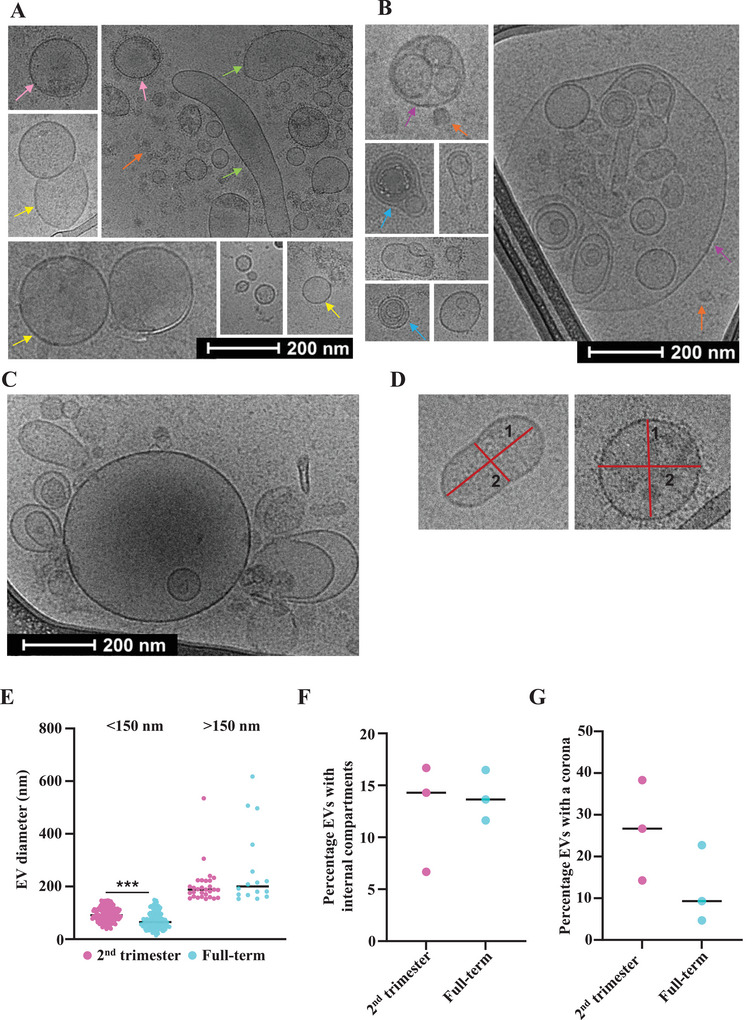
Amniotic fluid EVs have different morphologies. Cryo‐electron microscopy identified vesicles with (A) single‐membrane‐bound round (yellow arrows) or elongated (green arrows) shape, vesicles with corona (pink arrows), NVEPs (orange arrows), (B) multiple membranes (blue arrows) and/or multiple compartments (purple arrow), NVEPs (orange arrows). (C) Multiple EVs attached together with protein‐like structures were also visible. (D) EVs were measured in 2 perpendicular axes using ImageJ. Strip charts represent (E) the number of vesicles smaller and larger than 150 nm (each data point denotes a vesicle), (F) the percentage of vesicles with one or more internal compartments and (G) the number of vesicles with a corona, at each gestation time point. Statistical analysis used the Man–Whitney *U* test (E) and the Student's *t*‐test (F, G), with a confidence interval of 95%, in GraphPad Prism 10.1.0 (316). ***<0.001. NVEPs, non‐vesicular extracellular particles.

We visualised three AF‐EV samples from each group using cryo‐electron microscopy and identified different morphologies. Structures with a well‐defined dark line, indicating the lipid bilayer membrane, were considered as EVs (Emelyanov et al. 2020, Parra et al. 2024), to rule out reporting non‐vesicular extracellular particles (NVEPs) in our analyses (Welsh et al. 2024). Most EVs were simple, with the conventional morphology of single‐membrane‐bound round (yellow arrows) or elongated shapes (green arrows) (Figure 2A). EV corona was visible on some vesicles (pink arrows) (Figure 2A). This layer on the outside of the EV membrane represents the collection of proteins, lipids, carbohydrates and nucleic acids a vesicle acquires from its environment (Welsh et al. 2024, Yerneni et al. 2022, Wolf et al. 2022). While some EVs had multiple membranes (blue arrows), others had multiple compartments (purple arrows) (Figure 2B), which are relatively under‐represented EV morphologies in the literature (Broad et al. 2023). Orange arrows point to the NVEPs (Figure 2A,B), which are likely lipoprotein particles (Yuana et al. 2013). We also observed multiple EVs attached with presumed membrane proteins (yellow arrows) (Figure 2C), likely side effects of ultracentrifugation (Broad et al. 2023, Issman et al. 2013).

EV size is represented as the average measurement of the major and minor axes (Figure 2D). Figure 2E represents the number of EVs for each gestational age, categorised into two groups based on size: smaller than 150 nm and larger than 150 nm. The significant difference in the number of EVs smaller than 150 nm between gestation time points complemented the pattern observed in nanoparticle tracking analysis (Figure 1C,D).

We manually curated and counted the EVs with different features. Approximately 15% of all EVs had single or multiple compartments internal to the lipid bilayer membrane (Figure 2F). Approximately 25% of 2nd‐trimester and 10% of term EVs had a corona (Figure 2G).

### 3.4 Amniotic Fluid EV Proteome Varies in a Gestational Age‐Dependent Manner

**FIGURE 3:**
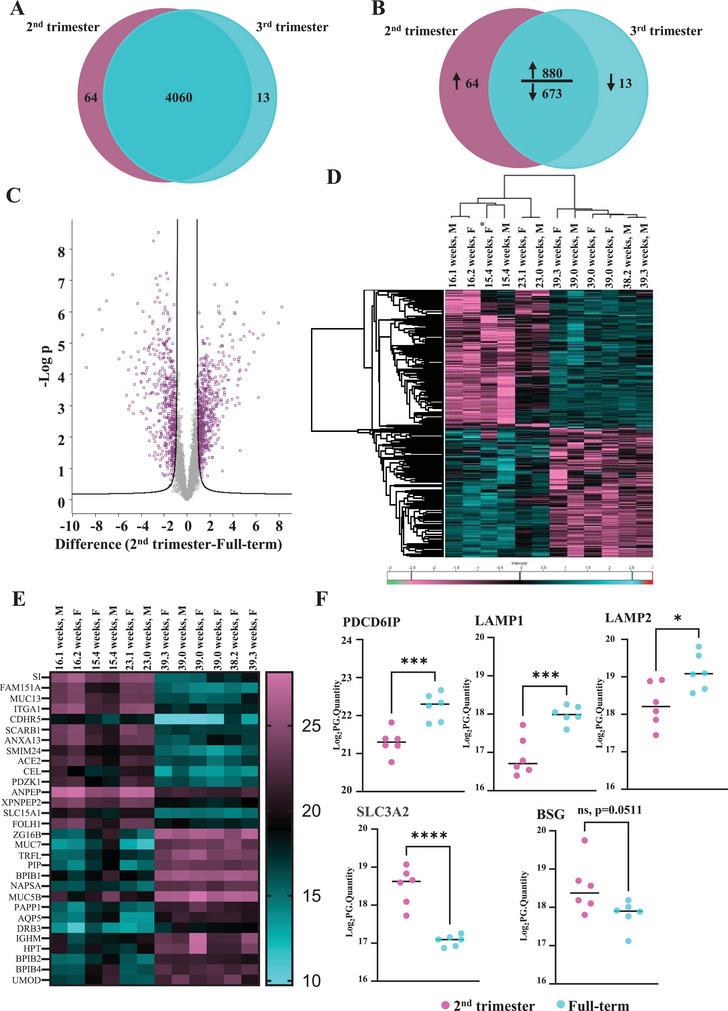
Amniotic fluid EV proteome changes according to gestational age. (A) Tandem mass spectrometry identified a total of 4137 proteins, with 64 proteins unique to the second trimester and 13 proteins unique to term AF‐EVs. (B) A total of 880 proteins were upregulated, and 673 were downregulated in second‐trimester AF‐EVs compared to term ones. (C) A total of 1099 proteins were significantly differentially enriched between the two gestational time points (FDR:0.05, S_0_:2). Heatmaps represent (D) the normalised log_2_ transformed values for the significantly differentially expressed proteins and (E) the 30 most statistically significant differentially expressed proteins. (F) Protein enrichment of exosome‐specific markers PDCD6IP (Alix), LAMP1 and LAMP2 and ectosome‐specific markers SLC3A2 and BSG as detected in mass spectrometry. (C, D and E) Statistical tests were performed on Perseus using unpaired, 2‐tailed Student's *t*‐test with FDR of 0.05 and 250 randomisations. (F) Graphs and statistics (unpaired, 2‐tailed Student's *t*‐test) using GraphPad Prism. *<0.05, **<0.01, ***<0.001, ****<0.0001.

Using a label‐free proteomics approach, we identified a total of 4137 proteins (Figure 3A). There were 64 proteins uniquely detected (present in all 6 replicates) in the second trimester and 13 in the term AF‐EVs. Tables 2, 3, and Table S1 represent these proteins and their potential involvement in fetal growth, according to published literature available on PubMed. Some common functions implicated with second‐trimester enriched proteins were cell proliferation, differentiation, angiogenesis and neurodevelopment. These proteins were also involved in cell signalling pathways associated with development, such as MAPK, PI3K/Akt, NF‐kB and Wnt (Table 2 and Table S1).

**TABLE 2:** The physiological role of the most abundant 15 proteins exclusive to the second‐trimester group.

Gene symbol	Biological pathways and potential role in human growth and development
RBP2	Retinoic acid signalling, uptake and metabolism of retinoids (Blaner et al. 2020), potential role in enteric nervous system development (Gao et al. 2021)
TM4SF20	Cell motility and adhesion (Fu et al. 2020), influence neural and craniofacial development (Wiszniewski et al. 2013)
ASAH2	Modulation of PI3K/Akt signalling pathway through ceramide metabolism (Terragni et al. 2008), regulates neuronal differentiation and cell cycle (Tanaka et al. 2012)
REG4	Cell proliferation and differentiation (Zheng et al. 2022), particularly in the gastrointestinal tract (Hartupee et al. 2001)
ADGRG7	G protein‐coupled receptor activity (Bjarnadóttir et al. 2004), potential critical function in angiogenesis, organogenesis, neurodevelopment and myelination (Folts et al. 2019)
UNC93A	May have a role in sensing nutrient limitation, cellular energy levels and autophagy, mammalian expression in brain and peripheral tissues (Ceder et al. 2017)
SERPINI2	Involved in insulin sensitivity, fibrinolysis and inflammatory response (Sánchez‐Navarro et al. 2021), potential role in pathways controlling eye growth and development (Janciauskiene et al. 2024), serpin‐loaded EVs used to promote wound healing in mice (Park et al. 2022)
SLC17A2	Transport of organic anions, involved in vesicular storage of the neurotransmitter and urate metabolism (Reimer 2013)
SULT1E1	Oestrogen homeostasis (Yi et al. 2021), regulation of hormonal signalling pathways during fetal neurodevelopment (Clarke et al. 2022)
IL18R1	Crucial role in immune system development (Ihim et al. 2022), angiogenesis (Bourdiec et al. 2016)
NUDT12	Regulation of nucleotide ratios according to changing conditions (Abdelraheim et al. 2003), mutations may cause intellectual disability and polyneuropathy (Diaz et al. 2020)
GRM2	Glutamate receptor signalling regulates MAPK/ERK1/2 signalling pathway, mediates neurotransmission and influences the development and integration of neurons (Ma et al. 2023)
GLTPD2	Involved in protein glycolipid transfer (Mishra et al. 2020), upregulated during necrotising enterocolitis (Jung et al. 2015)
NLRP6	Regulates caspase‐1, NF‐kB and MAPK signalling (Grenier et al. 2002, Anand et al. 2012), inflammasome formation and host defence against microorganisms (Levy et al. 2017)
CELA3B	A serine protease secreted by the pancreas (Basile et al. 2023)

*Note*: This table summarises the 15 most abundant of the 64 unique proteins in the second trimester and their potential roles in human physiology, growth and development. The rest of the unique proteins for the second trimester are listed in Table S1.

**TABLE 3:** The physiological role of the proteins exclusive to the term gestation group.

Gene symbol	Biological pathways and potential role in human growth and development
BPIFA2	Involved in innate immunity, antibacterial defence (Prokopovic et al. 2014, Geetha et al. 2003), salivary defence and surfactant properties crucial for oral health (Prokopovic et al. 2014)
TMEM52B	Regulates cell growth, proliferation and differentiation through EGFR signalling pathway (Deguen et al. 2016), high expression in the kidney (Xie et al. 2016), mutations may result in congenital heart defects, orofacial clefts and neural tube defects (Deguen et al. 2016)
HLA‐DPA1	Antigen presentation and immune response (Sugawara et al. 1994), potential role in maternal‐fetal immune tolerance (Wang et al. 2020)
GKN1	Exclusively and abundantly made in the stomach. Gastric mucosal defence and homeostasis (Dokhaee et al. 2018, Overstreet et al. 2021)
SLC39A8	Congenital mutations may result in abnormalities, including stunted growth, smaller‐sized liver, kidney, lung and cerebrum (Nebert and Liu 2019)
SUCNR1	Metabolic sensing, inflammation (Colombo et al. 2013), haematopoiesis in the bone marrow (Hakak et al. 2009), influences placental function (Atallah et al. 2021), lipolysis inhibition in adipose tissue (An et al. 2021)
MIA3	Protein trafficking (McCaughey et al. 2019), collagen synthesis (Wilson et al. 2011), important for extracellular matrix organisation, impacting fetal tissue development
PTPN21	Regulates cell adhesion and migration (Carlucci et al. 2008), and signalling pathways crucial for cellular growth and differentiation (Cho et al. 2017)
CAVIN3	Caveolae formation (Kovtun et al. 2015), regulation of muscle cell function and development, potentially impacts cardiovascular development (Insel and Patel 2009)
MENT	Chromatin organisation, structure and gene expression regulation (Grigoryev and Woodcock 1998)
HHIP	Hedgehog signalling pathway (Briscoe and Thérond 2013), essential for tissue patterning and lung development (Zeng et al. 2022)
SNAPC4	Transcription regulation (Frost et al. 2023)
ARHGAP29	Involved in Rho GTPase signalling (Saras et al. 1997), important for cell shape and movement, craniofacial development (Paul et al. 2017)

*Note*: This table summarises the EV proteins unique to the term gestation (Liu et al. 2008) and their potential role in human physiology, growth and development.

At term, the unique proteins were primarily responsible for immunity and defence, while some proteins corresponded to the regulation of cell proliferation and tissue patterning, implying controlled growth (Table 3). Among commonly detected proteins, 880 were highly abundant, and 673 were sparse in the second‐trimester AF‐EVs compared to term (Figure 3B).

We identified 1090 proteins that were significantly differentially enriched between the two gestational time points (Figure 3C) (false discovery rate of 0.05 and S_0_ = 2). Figure 3D shows the heatmap with unsupervised hierarchical clustering of proteins that are significantly differentially enriched. The proteins are arranged into two distinct main hierarchical clusters in this heatmap, indicating the clear separation of these proteins between the two gestation time points. The protein signatures of the first four samples corresponding to the early second trimester (15–16 weeks) clustered more closely. The clustering gradually changed towards the fifth and sixth samples, representing late second‐trimester gestations (23 weeks), before the signature completely changed in the term samples (38–39 weeks).

The group of 30 proteins with the highest fold changes are indicated in Figure 3E. The first 15 proteins were highly abundant in the second trimester‐derived AF‐EVs and were identified with terms such as carbohydrate metabolism (SI) (Jacob et al. 2000), cell adhesion (ITGA1) (Li et al. 2020) and (CDHR5) (Gray et al. 2021), lipid transport (SCARB1) (Shen et al. 2018) and peptide transport (SLC15A1) (Smith et al. 2013). The following 15 proteins were highly abundant in term‐derived AF‐EVs. Many of them, such as PIP (Umadat et al. 2013), UMOD (Jian et al. 2015), HLA‐DRB3 (Liu et al. 2021), BPIFB4 (Villa et al. 2015) and IGHM (Liu et al. 2019), were implicated in the immune response. MUC7 is mainly present in saliva and the respiratory tract (McGuckin et al. 2015), while AQP2 contributes to saliva secretion (Delporte et al. 2016).

Proteomic data showed significant enrichment of the ESCRT‐accessory protein PDCD6IP (Alix) in term EVs (Figure 3F), consistent with Western blotting (Figure 1A,B). Additionally, exosome‐exclusive markers LAMP1 and LAMP2 were also significantly enriched in term EVs. (Mathieu et al. 2021). Ectosome‐exclusive marker SLC3A2 (Mathieu et al. 2021) was significantly enriched in the second‐trimester AF‐EVs. BSG (Mathieu et al. 2021), another ectosome‐specific marker, followed the trend (*p* = 0.0511) These findings support the enrichment of the exosome fraction in the term.

### 3.5 Amniotic Fluid EV Proteome Is a Representation of Fetal Growth and Maturation

**FIGURE 4:**
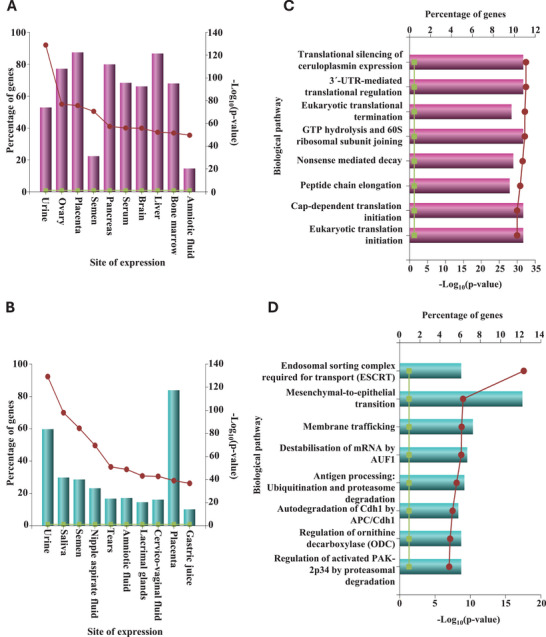
Amniotic fluid EV proteome represents gestational age‐dependent fetal growth and development. Enrichment analysis (Funrich) of proteins uniquely expressed and significantly abundant in (A) second trimester and (B) term were matched with distinct site‐specific protein signatures using UniProt, HPRD, Human Protein Atlas, Human Proteome Browser, Human Proteome Map, ProteomicsDB and Human Proteinpedia as background data resources (Gebara et al. 2022). A similar analysis was conducted to identify biological pathways enriched in (C) the second trimester and (D) term, using a combination of databases including Reactome, National Cancer Institute Data Catalogue, Cell map, HumanCyc and NCI‐Nature Pathway Interaction Database (Pathan et al. 2017).

We subsequently performed an enrichment analysis on the significantly abundant proteins identified for each gestational age, using the protein enrichment tool, FunRich (Pathan et al. 2017). Proteins enriched in the second‐trimester AF‐EVs were matched for expression in organs such as the ovary, pancreas, brain, liver and bone marrow (Figure 4A). AF‐EV proteome at term demonstrated a significant similarity to other human body fluids such as saliva, tears, cervicovaginal fluid and gastric juice (Figure 4B). Both gestational time points indicated a significant enrichment of placental and urine proteins.

The enrichment analysis for biological pathways matched second‐trimester‐enriched AF‐EV proteins with translation regulation, ribosome activity and protein synthesis‐related activities (Figure 4C), indicating the development the fetus undergoes during the second trimester.

Proteins enriched in the term AF‐EVs showed a significant involvement with biological processes that are important for bringing the fetal development phase to a halt (Figure 4D). Epithelial‐to‐mesenchymal transition (EMT) is a cellular signalling pathway instrumental in morphogenesis and organogenesis during fetal development (Kalluri and Weinberg 2009, Kim et al. 2014). The reverse process, mesenchymal‐to‐epithelial transition (MET), helps to generate somatic cells (Pei et al. 2019). We observed the activation of the MET pathway at term gestation. APC/Cdh1 complex, ornithine decarboxylase and active PAK2p34 all play essential roles in mitosis (Qiao et al. 2010), cell growth and differentiation (Jakobi et al. 2003) and cell survival (Clifford et al. 1995), respectively. The regulation of these proteins towards term gestation indicates the easing of the fetal development phase. Moreover, adaptive immunity activation towards the end of gestation may be implied in ‘antigen processing via ubiquitination and proteasomal degradation’ as the fetus approaches birth.

We detected a significantly high number of proteins associated with the Endosomal Sorting Complex Required for Transport (ESCRT) machinery in term AF‐EVs. This is a signalling pathway and the critical regulator of multivesicular body (MVB) formation and protein sorting into EVs (Schmidt and Teis 2012, Henne et al. 2011). This observation explains the enrichment of exosomes in the term AF‐EV pellet compared to that of the second trimester, as observed in Figures 1A,B, and 3F.

### 3.6 Proteomics Results After Excluding the Fetus With a Congenital Anomaly

Due to the presence of one sample with a congenital anomaly, we repeated the proteomics analysis for five pairs after excluding the fetus with the bilateral cleft/palate and the matched control. We observed a similar overall expression profile, with nine of the ten protein enrichment sites overlapping in the second trimester (Figure S3A and Figure 4A) and term (Figure S3B and Figure 4B. Similarly, the biological pathways implicated in the second‐trimester (Figure 4C and Figure S3C) and term (Figure 4D and Figure S3D) enriched proteins remained unchanged between the original and smaller cohorts. Therefore, we concluded that retaining these samples in our primary analysis was reasonable.

## 4 Discussion

This study presents evidence that EV biogenesis and secretion in the fetoplacental unit experience notable changes throughout gestation, mirroring the developmental maturation and changing physiology of the fetus. By isolating and characterising the small EV fraction, comprising both exosomes and small ectosomes, we observed a distinct shift in the predominant EV subtype between second‐trimester and term pregnancies. Specifically, second‐trimester AF‐EVs were enriched in ectosomes, which bud directly from the cell membrane, while term AF‐EVs were predominantly exosomes, originating from the endocytic pathway. This shift in EV subtype, correlated with significant differences in vesicle diameter, underscores the likelihood that these vesicles have unique biological roles at different stages of fetal development. Additionally, the biogenesis and secretion of ectosomes are generally considered less energy‐intensive than the formation of exosomes. This distinction arises because ectosome formation bypasses the complex intracellular trafficking and membrane fusion events required for exosome production in the endocytic pathway (Cocucci and Meldolesi 2015, Yu et al. 2023). Similarly, fetal development during the second trimester is characterised by organ development, where the energy consumption is lower compared to the last 8 weeks of pregnancy when the oxygen and nutrient demand increase (Mullis and Tonella 2008, Bauer et al. 1998). As such, the fetoplacental unit may prioritise less energy‐intensive ectosomes in the second trimester and more energy‐demanding exosomes towards term gestation. These findings enhance our understanding of the gestational age‐dependent characteristics of AF‐EVs and open new avenues for exploring their functional implications in fetal physiology.

This study is the first to conduct near‐native imaging of AF‐EVs with cryo‐electron microscopy, revealing their complicated morphologies for the first time. Similar EV phenotypes have been observed in cerebrospinal fluid (Emelyanov et al. 2020, Kurtjak et al. 2022), human ejaculate (Höög and Lötvall 2015), adipose tissue (Miroshnikova et al. 2023) and neuronal sources (Matthies et al. 2020). To differentiate these highly diverse EVs from other non‐EV artefacts, immunogold labelling may be applied in future cryo‐TEM studies (Malenica et al. 2021). It may be speculated that multi‐lamellar and multi‐vesicular structures are caused by ultracentrifugation‐based isolation protocols, such as in the current study, where smaller EVs may be forced into larger ones due to the force applied in high‐speed centrifugation (Broad et al. 2023, Issman et al. 2013). However, some disagree (Matthies et al. 2020). Others have speculated that these phenotypes are not mere artefacts of an isolation method but may have biological implications (Saadeldin et al. 2023). While multiple membranes are shown to contribute to the structural integrity of EVs (Kurtjak et al. 2022), multi‐EV clustering may indicate potential functional microdomains (Matthies et al. 2020). Further research may clarify the cause and role of these EV phenotypes.

We also observed corona on a substantial proportion of AF‐EVs. The corona is a recently described phenomenon in EV biology, comprising a complex conglomeration of biomolecules attached to the outer membrane. Coronas form after EV secretion into the extracellular environment and can alter EV functionality and, therefore, contribute to its biological properties (Wolf et al. 2022, Buzas 2022). Evidence from artificial nanoparticle research demonstrates multiple influences on corona formation, including physical parameters (vesicle diameter, surface curvature and zeta potential) and biochemical parameters (surface proteome) (Wolf et al. 2022, Oh et al. 2018, Lane 2020). Our results provide the first evidence of this phenomenon in human AF.

Our proteomics analysis revealed the gestational age‐dependent evolution of the protein cargo in AF‐EVs. We observed distinct differences in the protein signatures of second‐trimester and term AF‐EV samples. There were also detectable differences between the protein cargo of early (15–16 weeks) and late (23 weeks) stages of the second trimester (Figure 3D). Second‐trimester AF‐EVs showed a high abundance of proteins that drive active cell growth, differentiation and organ development. In contrast, term AF‐EVs contained proteins predominantly responsible for immunity, lung function and digestion. This shift in protein expression pattern reflects the fetus's known developmental trajectory across gestation. While we notice differences in EV cargo between mid‐gestation and term, the causal drivers of these differences remain uncertain. As we only provide snapshots at two time points (second trimester and full‐term), we cannot directly observe the dynamic changes throughout the entire gestation or identify the triggers for changes in EV release. For example, the term AF samples were collected before the onset of labour. The initiation of labour may have altered the molecular composition of the EVs (Radnaa et al. 2021, Dixon et al. 2018).

In previous studies, our group and others have explored AF cell‐free RNA to understand fetal development via a transcriptomic approach (Hui et al. 2012, Tarca et al. 2020, Hui et al. 2022), revealing similar RNA enrichment patterns to those observed in the current study. EVs are known as key mediators of extracellular RNA transport (Kim et al. 2023). This consistency with the AF cell‐free RNA literature supports our findings and offers further insights into the biology of AF cell‐free nucleic acids.

One sample of the second‐trimester group had a congenital anomaly. Given the discovery‐driven nature of this study and the similarity of the proteomics findings with and without this sample, we did not exclude it from analyses. However, we acknowledge that some fetuses with and without congenital anomalies may have different AF‐EV biology, which provides avenues for further research.

We have shown that AF contains EVs secreted from multiple cell types within the fetoplacental unit. AF and the EVs contained within it circulate throughout the fetal gastrointestinal, respiratory, pulmonary and urinary systems. Additionally, AF is in constant contact with the amniotic membranes and fetal surface of the placenta (Moore 2010, Cananzi et al. 2013). The presence of EVs in AF and their dynamic biology throughout gestation indicate their essential role in communication between fetal organ systems and the placenta. EVs are capable of docking on distant organs, releasing their biologically active cargo into the target cell's cytoplasm, thus influencing cellular signalling and metabolic processes of the recipient cell (Colombo et al. 2014, Cossetti et al. 2014). While AF‐EVs have been explored as biomarkers for pregnancy complications such as preeclampsia (Gebara et al. 2022), preterm labour (Dixon et al. 2018) and congenital cytomegalovirus infection (Bourgon et al. 2022), EV‐mediated cellular communication within the fetal compartment remains largely unexplored. Our characterisation of AF‐EVs contributes novel insights to this emerging field of research.

Our proteomic analysis uncovered gestation‐dependent protein enrichment patterns of AF‐EVs, emphasising the importance of considering gestational age when designing studies for biomarker discovery and therapeutic applications. For example, we have shown that while second‐trimester‐derived AF‐EVs are enriched in regenerative properties, term EVs are loaded with anti‐inflammatory molecules.

Some researchers responded to the restricted availability of amniocentesis samples by harvesting EVs from cultured stem/mesenchymal stromal cells derived from second‐trimester AF (Romani et al. 2015, Balbi et al. 2017, Tracy et al. 2019). While this is a convenient approach for large‐scale applications, it is unknown how the biological properties of EVs of cultured AF cells compare with those derived directly from AF. A comparative analysis of EVs derived from fresh AF versus cultured AF‐derived cells may provide further insights into their in vivo biology.

Due to the limited availability of second‐trimester amniotic fluid samples from uncomplicated pregnancies, we were limited to six samples per group. All donors were healthy and within a narrow maternal age range (Table 1). Despite our small sample size, our analyses revealed consistent and statistically significant results. However, we acknowledge that donor heterogeneity may have influenced the results andverifying our results in larger independent cohorts would further strengthen these findings.

Obtaining a pure population of EVs remains a challenge (Fonseka et al. 2021). Each purification consists of EVs originating from more than one subcellular origin, and therefore, our results represent a mixed EV population. These EVs may mainly originate from fetal sources (fetus, placenta and fetal membranes) and possibly some from maternal sources as well, due to the transplacental transfer of maternal EV (Farrelly et al. 2023), albeit in small amounts. Furthermore, employing ultracentrifugation solely for the collection of small EVs could have introduced certain impurities, such as NVEPs and protein contaminants (Welsh et al. 2024). These impurities may be minimised by utilising combination approaches such as centrifugation and size‐exclusion chromatography (Gebara et al. 2022) or sucrose‐density gradient centrifugation (Xie et al. 2017) in future studies. Additionally, analysing the 15K pellet may have widened our understanding of the EV‐borne biological information of AF.

Our functional analysis is likely affected by the literature bias inherent in proteomics. Information about fetal development‐related molecular mechanisms is sparse (Edlow et al. 2015). The analysis tools used in this study could not compare the current data set specifically against a fetal proteome data resource. It is also acknowledged that certain pathologies, such as cancer, neurodegenerative diseases and cardiovascular diseases, are widely researched and thus over‐represented in annotation databases (Robles et al. 2024, Deutsch et al. 2017). Therefore, in presenting the enrichment analysis, we omitted the results of unapplicable adult pathologies and transformed cell lines, focusing on the findings relevant to human fetal physiology.

## 5 Conclusions

In conclusion, AF‐EVs represent an intriguing mechanism for intercellular communication within and potentially outside the fetoplacental unit. This comprehensive investigation demonstrates the diverse morphology and dynamic biology of fetal EV secretion and function. The gestational age‐dependent enrichment patterns of EV proteins suggest a purposive nature to EV cargo packaging and the potential of AF‐EVs to provide new insights into fetal intercellular communication and physiology. Understanding the biological significance of the different predominant EV subtypes at various gestational stages is a crucial next step in this field.

## Author Contributions

**Ishara Atukorala**: methodology, investigation, validation, formal analysis, data curation, conceptualization, writing – original draft, writing – review and editing. **Sally Beard**: writing – review and editing, project administration. **Ching‐Seng Ang**: methodology, data curation, writing – review and editing. **Hamish Brown**: methodology, data curation, writing – review and editing. **Swetha Raghavan**: writing – review and editing, formal analysis, investigation. **Natasha de Alwis**: writing – review and editing, investigation. **Bianca Fato**: writing – review and editing, investigation. **Natalie Binder**: writing – review and editing, investigation. **Natalie Hannan**: resources, writing – review and editing. **Lisa Hui**: conceptualization, supervision, funding acquisition, resources, writing – review and editing.

## Ethics Statement

The Human Research Ethics Committee of Mercy Health (2023‐014) approved this study.

## Consent

AF samples were collected prospectively at Mercy Hospital for Women in Melbourne, Australia, following written, informed consent from the participants.

## Conflicts of Interest

The authors declare no conflicts of interest.

## Supporting information




**Supplementary Table**: jex270085‐sup‐0001‐SuppMat.docx

**Supplementary Material**: jex270085‐sup‐0002‐SuppMat.tsv

**Supplementary Figure 1 Pre‐ and post‐normalisation plots for the proteomic data**. Box and whisker plots showing the data distribution for the 12 samples, before and after normalisation, using automatic cross‐run normalisation in Spectronaut software (v. 17.5.230413.55965).

**Supplementary Figure 2 Sypro Ruby staining and densitometry of the complete Western blot**. Western blot was stained with Sypro Ruby blot stain and analysed using Image Lab software (Bio‐Rad). (A) The protein ladder is on the first lane, followed by term samples and second‐trimester samples. Please note that this Western blot was cropped to separate the 2 sample groups and switched sides to obtain Figure 1A. (B) Complete Western blot with probing for Alix. (C) Complete Western blot with probing for CD63 and CD9.

**Supplementary Figure 3 Protein enrichment analysis excluding the sample with a congenital anomaly**. Enrichment analysis (Funrich) performed in Figure 4 was repeated for five pairs, excluding the second‐trimester sample with bilateral cleft lip and palate and a fetal sex‐matched control. Proteins uniquely expressed and significantly abundant in (A) second trimester and (B) term were matched with distinct site‐specific protein signatures. Nine out of the ten sites enriched in both gestations overlapped with their original analyses results presented in Figures 4A and 4B. A similar analysis was conducted to identify biological pathways enriched in (C) the second trimester and (D) term. Compared to their original analysis, there were no changes in the enriched biological pathways at both gestations in this 5‐pair analysis.

## Data Availability

The data that support the findings of this study are openly available in PRIDE at http://www.ebi.ac.uk/pride, reference number PXD058882.
