# Supplementary material for: Amniotic Fluid Extracellular Vesicle Properties Evolve With Gestational Age and Reflect Fetal Development
Source: J Extracell Biol. 2025 Oct 8;4(10):e70085. doi: 10.1002/jex2.70085 (PMC12508267; doi:10.1002/jex2.70085)
Supplement: Supplementary file 1 — Supplementary Table: jex270085‐sup‐0001‐SuppMat.docx [file JEX2-4-e70085-s004.docx]

Supplementary Table 1 The physiological role of proteins uniquely expressed in the second trimester - an extension to Table 2

| **Gene symbol** | **Biological Pathways and potential role in human growth and development** |
| --- | --- |
| ZNFX1 | An interferon-stimulating gene, Regulates the IFN signalling pathway and innate immune response (29) |
| LIPC | Essential for the maintenance of lipid balance, which is crucial for cell membrane integrity, energy storage, and hormone production (30) |
| RTN4RL2 | Involved in the development and refinement of neural structures (31), Corpus callosum development and morphogenesis (32) |
| GPA33 | Hematopoietic stem cell maintenance, Tissue remodelling and organogenesis (33) |
| UNC5C | Critical role in neurodevelopment by directing axon extension, Guidance and cell migration (34) |
| P2RX1 | Important in neurogenesis, neurite outgrowth and central nervous system development (35) |
| PHOSPHO1 | Phospholipid homeostasis, biomineralisation of bone and other hard tissues (36) |
| ITGAL | Development of the immune system, particularly in T-cell development and function (37) and leukocyte adhesion and migration (38) |
| C2orf72 | Expressed in the placenta (39) |
| ADCY3 | Regulates the cAMP signalling in neuronal cilia (40), Loss of ADCY3 may lead to reduced neuronal activity and depression-like behaviour (41) |
| CHRNE | Neuromuscular junction signalling, Essential for the development of neuromuscular junctions, Crucial for muscle development and function (42) |
| MICAL3 | Critical for actin filament organization-related processes such as axonal growth and nerve growth cone motility (43) |
| CRHBP | Mediates signalling in the hypothalamic–pituitary–adrenal (HPA) axis (44) |
| LRRC75A | Potential role in angiogenesis (45) |
| CDH17 | Integrin-binding cell adhesion protein, Expressed in elevated levels in the fetal liver and gastrointestinal tract (46) |
| SLC17A1 | Anion transport molecule involved in urate excretion (47) |
| APOBEC2 | Functions in DNA repair and RNA editing, regulating cell reprogramming during development (48) |
| LRRC19 | Important in the development of the gut immune system and inflammation response (49) |
| LTBP4 | Regulates TGF-β signalling, involved in alveolar septation and blastogenesis during fetal development (50) |
| PRSS35 | Potential role in craniofacial growth, mutations cause cleft lip/palate (51) |
| HAAO | Part of the kynurenine pathway, Influences tryptophan metabolism, Important in neurodevelopment (52) |
| FGGY | Involved in Glycolysis and gluconeogenesis, Potential role in neuromuscular development (53) |
| COL9A1 | Extracellular matrix organisation (54), Important in the formation of cartilage and skeleton in early development (55) |
| SNX30 | Intracellular protein trafficking via endosome to the trans-Golgi network (TGN) pathway, Intracellular signalling (56) |
| SEMA6D | Semaphorin signalling, Important in vascular patterning and motor neuronal axon growth (57) |
| SLC5A11 | Regulates glucose levels (58), Potential role in the accumulation of nutrients, neurotransmitters and ions (59) |
| BDH1 | Butyrate metabolism, Synthesis and degradation of ketone bodies (60) |
| CXCL14 | Chemokine signalling, Promotes the development of neurons (61), Paracrine/autocrine-dependent regulation of trophoblast outgrowth (62) |
| PUM1 | Post-transcriptional regulation (63) |
| SGK3 | Stimulates Wnt signalling pathway, Involved in hair follicle morphogenesis (64) |
| SEMA6A | Maintenance of neuroendocrine homeostasis (65), Angiogenesis via VEGF signalling (66) |
| GFRA3 | Glial cell line-derived neurotrophic factor signalling, Important in neurogenesis, axon guidance, synaptogenesis and cardiogenesis (67) |
| GAMT | Creatine metabolism, Potential role in neurodevelopment, Deficiency may cause development delay, ataxia and seizures (68) |
| KDELR3 | Functions in the ER stress response, Important in melanocyte development (69) |
| ROR2 | Non-canonical Wnt signalling pathway, Development of the cardiovascular system (70) |
| GJC1 | Gap junction communication, Critical in fetal cardiac development, Mutations cause congenital heart disease (71) |
| CTH | Regulation of scavenging reactive oxygen species (72) |
| GLT8D2 | Potential role in triglyceride metabolism (73) |
| TMEM236 | Expression in the intestinal tissue (74) |
| MAPKBP1 | MAPK signalling, Potential role in the development of kidney, Mutations cause nephronophthisis (75) |
| IL4I1 | Interleukin signalling, Potential role in developing immune system (76) and microglia (77) |
| NLN | Neurotransmitter degradation, Potential role in cardiovascular and renal homeostasis (78) |
| MTARC2 | Involved in lipogenesis, Mitochondrial function (79) |
| PTGR3 | Prostaglandin metabolism, Critical modulator of adiposity (80) |
| TRIM14 | Regulates cell proliferation, migration, invasion, cell cycle progression and differentiation (81) |
| PRG4 | Regulates osteogenic smooth muscle cell differentiation, and vascular remodelling (82) |
| CRAT | Fatty acid metabolism, Expressed in fetal liver and brown adipose tissue (83) |
| MSTO1 | Mitochondrial dynamics and energy production, Mutations may cause early-onset myopathy and cerebellar ataxia (84) |
| ILVBL | Amino acid and pyruvate metabolism, Energy metabolism (85) |

*This table extends from Table 2, summarising 49 of the 64 unique proteins expressed in the second trimester and their potential role in human physiology, growth and development.*

1. Blaner WS, Brun PJ, Calderon RM, Golczak M. Retinol-binding protein 2 (RBP2): biology and pathobiology. Crit Rev Biochem Mol Biol. 2020;55(2):197-218.

2. Gao T, Wright-Jin EC, Sengupta R, Anderson JB, Heuckeroth RO. Cell-autonomous retinoic acid receptor signaling has stage-specific effects on mouse enteric nervous system. JCI Insight. 2021;6(10).

3. Fu F, Yang X, Zheng M, Zhao Q, Zhang K, Li Z, et al. Role of Transmembrane 4 L Six Family 1 in the Development and Progression of Cancer. Front Mol Biosci. 2020;7:202.

4. Wiszniewski W, Hunter JV, Hanchard NA, Willer JR, Shaw C, Tian Q, et al. TM4SF20 ancestral deletion and susceptibility to a pediatric disorder of early language delay and cerebral white matter hyperintensities. Am J Hum Genet. 2013;93(2):197-210.

5. Terragni J, Graham JR, Adams KW, Schaffer ME, Tullai JW, Cooper GM. Phosphatidylinositol 3-kinase signaling in proliferating cells maintains an anti-apoptotic transcriptional program mediated by inhibition of FOXO and non-canonical activation of NFκB transcription factors. BMC Cell Biology. 2008;9(1):6.

6. Tanaka K, Tamiya-Koizumi K, Hagiwara K, Ito H, Takagi A, Kojima T, et al. Role of down-regulated neutral ceramidase during all-trans retinoic acid-induced neuronal differentiation in SH-SY5Y neuroblastoma cells. The Journal of Biochemistry. 2012;151(6):611-20.

7. Zheng HC, Xue H, Zhang CY. REG4 promotes the proliferation and anti-apoptosis of cancer. Front Cell Dev Biol. 2022;10:1012193.

8. Hartupee JC, Zhang H, Bonaldo MF, Soares MB, Dieckgraefe BK. Isolation and characterization of a cDNA encoding a novel member of the human regenerating protein family: Reg IV. Biochim Biophys Acta. 2001;1518(3):287-93.

9. Bjarnadóttir TK, Fredriksson R, Höglund PJ, Gloriam DE, Lagerström MC, Schiöth HB. The human and mouse repertoire of the adhesion family of G-protein-coupled receptors. Genomics. 2004;84(1):23-33.

10. Folts CJ, Giera S, Li T, Piao X. Adhesion G Protein-Coupled Receptors as Drug Targets for Neurological Diseases. Trends Pharmacol Sci. 2019;40(4):278-93.

11. Ceder MM, Lekholm E, Hellsten SV, Perland E, Fredriksson R. The Neuronal and Peripheral Expressed Membrane-Bound UNC93A Respond to Nutrient Availability in Mice. Front Mol Neurosci. 2017;10:351.

12. Sánchez-Navarro A, González-Soria I, Caldiño-Bohn R, Bobadilla NA. An integrative view of serpins in health and disease: the contribution of SerpinA3. Am J Physiol Cell Physiol. 2021;320(1):C106-c18.

13. Janciauskiene S, Lechowicz U, Pelc M, Olejnicka B, Chorostowska-Wynimko J. Diagnostic and therapeutic value of human serpin family proteins. Biomedicine & Pharmacotherapy. 2024;175:116618.

14. Park DJ, Duggan E, Ho K, Dorschner RA, Dobke M, Nolan JP, et al. Serpin-loaded extracellular vesicles promote tissue repair in a mouse model of impaired wound healing. J Nanobiotechnology. 2022;20(1):474.

15. Reimer RJ. SLC17: a functionally diverse family of organic anion transporters. Mol Aspects Med. 2013;34(2-3):350-9.

16. Yi M, Negishi M, Lee SJ. Estrogen Sulfotransferase (SULT1E1): Its Molecular Regulation, Polymorphisms, and Clinical Perspectives. J Pers Med. 2021;11(3).

17. Clarke T, Fernandez FE, Dawson PA. Sulfation Pathways During Neurodevelopment. Front Mol Biosci. 2022;9:866196.

18. Ihim SA, Abubakar SD, Zian Z, Sasaki T, Saffarioun M, Maleknia S, et al. Interleukin-18 cytokine in immunity, inflammation, and autoimmunity: Biological role in induction, regulation, and treatment. Front Immunol. 2022;13:919973.

19. Bourdiec A, Ahmad SF, Lachhab A, Akoum A. Regulation of inflammatory and angiogenesis mediators in a functional model of decidualized endometrial stromal cells. Reprod Biomed Online. 2016;32(1):85-95.

20. Abdelraheim SR, Spiller DG, McLennan AG. Mammalian NADH diphosphatases of the Nudix family: cloning and characterization of the human peroxisomal NUDT12 protein. Biochem J. 2003;374(Pt 2):329-35.

21. Diaz F, Khosa S, Niyazov D, Lee H, Person R, Morrow MM, et al. Novel NUDT2 variant causes intellectual disability and polyneuropathy. Ann Clin Transl Neurol. 2020;7(11):2320-5.

22. Ma J, Hu Z, Yue H, Luo Y, Wang C, Wu X, et al. GRM2 Regulates Functional Integration of Adult-Born DGCs by Paradoxically Modulating MEK/ERK1/2 Pathway. The Journal of Neuroscience. 2023;43(16):2822.

23. Mishra SK, Gao YG, Zou X, Stephenson DJ, Malinina L, Hinchcliffe EH, et al. Emerging roles for human glycolipid transfer protein superfamily members in the regulation of autophagy, inflammation, and cell death. Prog Lipid Res. 2020;78:101031.

24. Jung K, Kim JH, Cheong HS, Shin E, Kim SH, Hwang JY, et al. Gene expression profile of necrotizing enterocolitis model in neonatal mice. Int J Surg. 2015;23(Pt A):28-34.

25. Grenier JM, Wang L, Manji GA, Huang W-J, Al-Garawi A, Kelly R, et al. Functional screening of five PYPAF family members identifies PYPAF5 as a novel regulator of NF-κB and caspase-1. FEBS Letters. 2002;530(1):73-8.

26. Anand PK, Malireddi RKS, Lukens JR, Vogel P, Bertin J, Lamkanfi M, et al. NLRP6 negatively regulates innate immunity and host defence against bacterial pathogens. Nature. 2012;488(7411):389-93.

27. Levy M, Shapiro H, Thaiss CA, Elinav E. NLRP6: A Multifaceted Innate Immune Sensor. Trends Immunol. 2017;38(4):248-60.

28. Basile G, Vetere A, Hu J, Ijaduola O, Zhang Y, Liu KC, et al. Excess pancreatic elastase alters acinar-β cell communication by impairing the mechano-signaling and the PAR2 pathways. Cell Metab. 2023;35(7):1242-60.e9.

29. Liu H, Han Z, Chen L, Zhang J, Zhang Z, Chen Y, et al. ZNFX1 promotes AMPK-mediated autophagy against Mycobacterium tuberculosis by stabilizing Prkaa2 mRNA. JCI Insight. 2024;9(1).

30. Liao YH, Er LK, Wu S, Ko YL, Teng MS. Functional Haplotype of LIPC Induces Triglyceride-Mediated Suppression of HDL-C Levels According to Genome-Wide Association Studies. Genes (Basel). 2021;12(2).

31. Wang J, Miao Y, Wicklein R, Sun Z, Wang J, Jude KM, et al. RTN4/NoGo-receptor binding to BAI adhesion-GPCRs regulates neuronal development. Cell. 2022;185(1):218.

32. Yoo S-W, Motari MG, Schnaar RL. Agenesis of the corpus callosum in Nogo receptor deficient mice. The Journal of comparative neurology. 2017;525(2):291-301.

33. Opstelten R, Suwandi JS, Slot MC, Morgana F, Scott AM, Laban S, et al. GPA33 is expressed on multiple human blood cell types and distinguishes CD4(+) central memory T cells with and without effector function. Eur J Immunol. 2021;51(6):1377-89.

34. Li Q, Wang BL, Sun FR, Li JQ, Cao XP, Tan L. The role of UNC5C in Alzheimer's disease. Ann Transl Med. 2018;6(10):178.

35. Baines KJ, Hillier DM, Haddad FL, Rajakumar N, Schmid S, Renaud SJ. Maternal Immune Activation Alters Fetal Brain Development and Enhances Proliferation of Neural Precursor Cells in Rats. Front Immunol. 2020;11:1145.

36. Dillon S, Staines KA, Millán JL, Farquharson C. How To Build a Bone: PHOSPHO1, Biomineralization, and Beyond. JBMR Plus. 2019;3(7):e10202.

37. Zhang J, Teh M, Kim J, Eva MM, Cayrol R, Meade R, et al. A Loss-of-Function Mutation in the Integrin Alpha L (Itgal) Gene Contributes to Susceptibility to Salmonella enterica Serovar Typhimurium Infection in Collaborative Cross Strain CC042. Infect Immun. 2019;88(1).

38. Li R, Wu X, Xue K, Li J. ITGAL infers adverse prognosis and correlates with immunity in acute myeloid leukemia. Cancer Cell Int. 2022;22(1):268.

39. Robles J, Prakash A, Vizcaíno JA, Casal JI. Integrated meta-analysis of colorectal cancer public proteomic datasets for biomarker discovery and validation. PLoS Comput Biol. 2024;20(1):e1011828.

40. Qiu L, LeBel RP, Storm DR, Chen X. Type 3 adenylyl cyclase: a key enzyme mediating the cAMP signaling in neuronal cilia. Int J Physiol Pathophysiol Pharmacol. 2016;8(3):95-108.

41. Chen X, Luo J, Leng Y, Yang Y, Zweifel LS, Palmiter RD, et al. Ablation of Type III Adenylyl Cyclase in Mice Causes Reduced Neuronal Activity, Altered Sleep Pattern, and Depression-like Phenotypes. Biol Psychiatry. 2016;80(11):836-48.

42. Ohkawara B, Ito M, Ohno K. Secreted Signaling Molecules at the Neuromuscular Junction in Physiology and Pathology. Int J Mol Sci. 2021;22(5).

43. Giridharan SS, Caplan S. MICAL-family proteins: Complex regulators of the actin cytoskeleton. Antioxid Redox Signal. 2014;20(13):2059-73.

44. Chen B, Chen S, Wang X, Zhang J, Wang H, Li J, et al. A pan-cancer analysis uncovering the function of CRHBP in tumor immunity, prognosis and drug response: especially its function in LIHC. Scientific Reports. 2024;14(1):3112.

45. Miura T, Kouno T, Takano M, Kuroda T, Yamamoto Y, Kusakawa S, et al. Single-Cell RNA-Seq Reveals LRRC75A-Expressing Cell Population Involved in VEGF Secretion of Multipotent Mesenchymal Stromal/Stem Cells Under Ischemia. Stem Cells Translational Medicine. 2023;12(6):379-90.

46. Bartolomé RA, Peláez-García A, Gomez I, Torres S, Fernandez-Aceñero MJ, Escudero-Paniagua B, et al. An RGD Motif Present in Cadherin 17 Induces Integrin Activation and Tumor Growth*. Journal of Biological Chemistry. 2014;289(50):34801-14.

47. Iharada M, Miyaji T, Fujimoto T, Hiasa M, Anzai N, Omote H, et al. Type 1 Sodium-dependent Phosphate Transporter (SLC17A1 Protein) Is a Cl−-dependent Urate Exporter*. Journal of Biological Chemistry. 2010;285(34):26107-13.

48. Pecori R, Di Giorgio S, Paulo Lorenzo J, Nina Papavasiliou F. Functions and consequences of AID/APOBEC-mediated DNA and RNA deamination. Nature Reviews Genetics. 2022;23(8):505-18.

49. Cao S, Su X, Zeng B, Yan H, Huang Y, Wang E, et al. The Gut Epithelial Receptor LRRC19 Promotes the Recruitment of Immune Cells and Gut Inflammation. Cell Rep. 2016;14(4):695-707.

50. Dabovic B, Chen Y, Choi J, Vassallo M, Dietz HC, Ramirez F, et al. Dual functions for LTBP in lung development: LTBP-4 independently modulates elastogenesis and TGF-beta activity. J Cell Physiol. 2009;219(1):14-22.

51. Letra A, Menezes R, Fonseca RF, Govil M, McHenry T, Murphy MJ, et al. Novel cleft susceptibility genes in chromosome 6q. J Dent Res. 2010;89(9):927-32.

52. Higazi AM, Kamel HM, Abdel-Naeem EA, Abdullah NM, Mahrous DM, Osman AM. Expression analysis of selected genes involved in tryptophan metabolic pathways in Egyptian children with Autism Spectrum Disorder and learning disabilities. Sci Rep. 2021;11(1):6931.

53. Smith AL, Gjoka E, Izhar M, Novo KJ, Mason BC, De Las Casas A, et al. FGGY carbohydrate kinase domain containing is expressed and alternatively spliced in skeletal muscle and attenuates MAP kinase and Akt signaling. Gene. 2021;800:145836.

54. Arseni L, Lombardi A, Orioli D. From Structure to Phenotype: Impact of Collagen Alterations on Human Health. Int J Mol Sci. 2018;19(5).

55. MedlinePlus. COL9A1 gene Bethesda (MD): National Library of Medicine (US); 2020 [updated Jun 24, 2020. Available from: <https://medlineplus.gov/about/using/citation/>.

56. Vieira N, Rito T, Correia-Neves M, Sousa N. Sorting Out Sorting Nexins Functions in the Nervous System in Health and Disease. Molecular Neurobiology. 2021;58(8):4070-106.

57. Sheng J, Xu J, Geng K, Liu D. Sema6D Regulates Zebrafish Vascular Patterning and Motor Neuronal Axon Growth in Spinal Cord. Front Mol Neurosci. 2022;15:854556.

58. Ugrankar R, Theodoropoulos P, Akdemir F, Henne WM, Graff JM. Circulating glucose levels inversely correlate with Drosophila larval feeding through insulin signaling and SLC5A11. Communications Biology. 2018;1(1):110.

59. Roll P, Massacrier A, Pereira S, Robaglia-Schlupp A, Cau P, Szepetowski P. New human sodium/glucose cotransporter gene (KST1): identification, characterization, and mutation analysis in ICCA (infantile convulsions and choreoathetosis) and BFIC (benign familial infantile convulsions) families. Gene. 2002;285(1):141-8.

60. Muroya S, Zhang Y, Kinoshita A, Otomaru K, Oshima K, Gotoh Y, et al. Maternal Undernutrition during Pregnancy Alters Amino Acid Metabolism and Gene Expression Associated with Energy Metabolism and Angiogenesis in Fetal Calf Muscle. Metabolites. 2021;11(9).

61. Zhang Y, Jin Y, Li J, Yan Y, Wang T, Wang X, et al. CXCL14 as a Key Regulator of Neuronal Development: Insights from Its Receptor and Multi-Omics Analysis. International Journal of Molecular Sciences [Internet]. 2024; 25(3).

62. Kuang H, Chen Q, Fan X, Zhang Y, Zhang L, Peng H, et al. CXCL14 inhibits trophoblast outgrowth via a paracrine/autocrine manner during early pregnancy in mice. J Cell Physiol. 2009;221(2):448-57.

63. Uyhazi KE, Yang Y, Liu N, Qi H, Huang XA, Mak W, et al. Pumilio proteins utilize distinct regulatory mechanisms to achieve complementary functions required for pluripotency and embryogenesis. Proc Natl Acad Sci U S A. 2020;117(14):7851-62.

64. Mauro TM, McCormick JA, Wang J, Boini KM, Ray L, Monks B, et al. Akt2 and SGK3 are both determinants of postnatal hair follicle development. Faseb j. 2009;23(9):3193-202.

65. Lettieri A, Oleari R, van den Munkhof MH, van Battum EY, Verhagen MG, Tacconi C, et al. SEMA6A drives GnRH neuron-dependent puberty onset by tuning median eminence vascular permeability. Nature Communications. 2023;14(1):8097.

66. Ohnuki H, Tosato G. Characterization of Semaphorin 6A-Mediated Effects on Angiogenesis Through Regulation of VEGF Signaling. Methods Mol Biol. 2017;1493:345-61.

67. Airaksinen MS, Saarma M. The GDNF family: Signalling, biological functions and therapeutic value. Nature Reviews Neuroscience. 2002;3(5):383-94.

68. Sun Q. Chapter 14 - Cerebral creatine deficiency syndromes. In: Garg U, Smith LD, editors. Biomarkers in Inborn Errors of Metabolism. San Diego: Elsevier; 2017. p. 331-41.

69. Marie KL, Sassano A, Yang HH, Michalowski AM, Michael HT, Guo T, et al. Melanoblast transcriptome analysis reveals pathways promoting melanoma metastasis. Nat Commun. 2020;11(1):333.

70. Guo R, Xing QS. Roles of Wnt Signaling Pathway and ROR2 Receptor in Embryonic Development: An Update Review Article. Epigenet Insights. 2022;15:25168657211064232.

71. Li YJ, Wang J, Ye WG, Liu XY, Li L, Qiu XB, et al. Discovery of GJC1 (Cx45) as a New Gene Underlying Congenital Heart Disease and Arrhythmias. Biology (Basel). 2023;12(3).

72. Wang M, Guo Z, Wang S. Cystathionine gamma-lyase expression is regulated by exogenous hydrogen peroxide in the mammalian cells. Gene Expr. 2012;15(5-6):235-41.

73. Zhan Y, Zhao F, Xie P, Zhong L, Li D, Gai Q, et al. Mechanism of the effect of glycosyltransferase GLT8D2 on fatty liver. Lipids Health Dis. 2015;14:43.

74. Maurya NS, Kushwaha S, Chawade A, Mani A. Transcriptome profiling by combined machine learning and statistical R analysis identifies TMEM236 as a potential novel diagnostic biomarker for colorectal cancer. Scientific Reports. 2021;11(1):14304.

75. Schönauer R, Jin W, Ertel A, Nemitz-Kliemchen M, Panitz N, Hantmann E, et al. Novel nephronophthisis-associated variants reveal functional importance of MAPKBP1 dimerization for centriolar recruitment. Kidney Int. 2020;98(4):958-69.

76. Sadik A, Somarribas Patterson LF, Öztürk S, Mohapatra SR, Panitz V, Secker PF, et al. IL4I1 Is a Metabolic Immune Checkpoint that Activates the AHR and Promotes Tumor Progression. Cell. 2020;182(5):1252-70.e34.

77. Liu W, Dong C, Liu X. [The role of IL4I1 in immunoregulation: An update]. Xi Bao Yu Fen Zi Mian Yi Xue Za Zhi. 2021;37(1):79-83.

78. Cavalcanti DM, Castro LM, Rosa Neto JC, Seelaender M, Neves RX, Oliveira V, et al. Neurolysin knockout mice generation and initial phenotype characterization. J Biol Chem. 2014;289(22):15426-40.

79. Neve EP, Köfeler H, Hendriks DF, Nordling Å, Gogvadze V, Mkrtchian S, et al. Expression and Function of mARC: Roles in Lipogenesis and Metabolic Activation of Ximelagatran. PLoS One. 2015;10(9):e0138487.

80. Yu YH, Chang YC, Su TH, Nong JY, Li CC, Chuang LM. Prostaglandin reductase-3 negatively modulates adipogenesis through regulation of PPARγ activity. J Lipid Res. 2013;54(9):2391-9.

81. Xu G, Guo Y, Xu D, Wang Y, Shen Y, Wang F, et al. TRIM14 regulates cell proliferation and invasion in osteosarcoma via promotion of the AKT signaling pathway. Scientific Reports. 2017;7(1):42411.

82. Seime T, Akbulut AC, Liljeqvist ML, Siika A, Jin H, Winski G, et al. Proteoglycan 4 Modulates Osteogenic Smooth Muscle Cell Differentiation during Vascular Remodeling and Intimal Calcification. Cells. 2021;10(6).

83. Seccombe DW, Hahn P. Carnitine acetyltransferase in developing mammals. Biol Neonate. 1980;38(1-2):90-5.

84. Chen J, Xiao J, Chen G, Xu Q, Wu X, Tian L, et al. Indentification of novel MSTO1 compound heterozygous mutations in a Chinese family with recessive cerebellar atrophy and ataxia. Front Neurol. 2022;13:988519.

85. Chang HS, Park JS, Lee HS, Lyu J, Son JH, Choi IS, et al. Association analysis of ILVBL gene polymorphisms with aspirin-exacerbated respiratory disease in asthma. BMC Pulm Med. 2017;17(1):210.
