# Supplementary figures and images for: Amniotic Fluid Extracellular Vesicle Properties Evolve With Gestational Age and Reflect Fetal Development
Source: J Extracell Biol. 2025 Oct 8;4(10):e70085. doi: 10.1002/jex2.70085 (PMC12508267; doi:10.1002/jex2.70085)

Supplementary figure 1

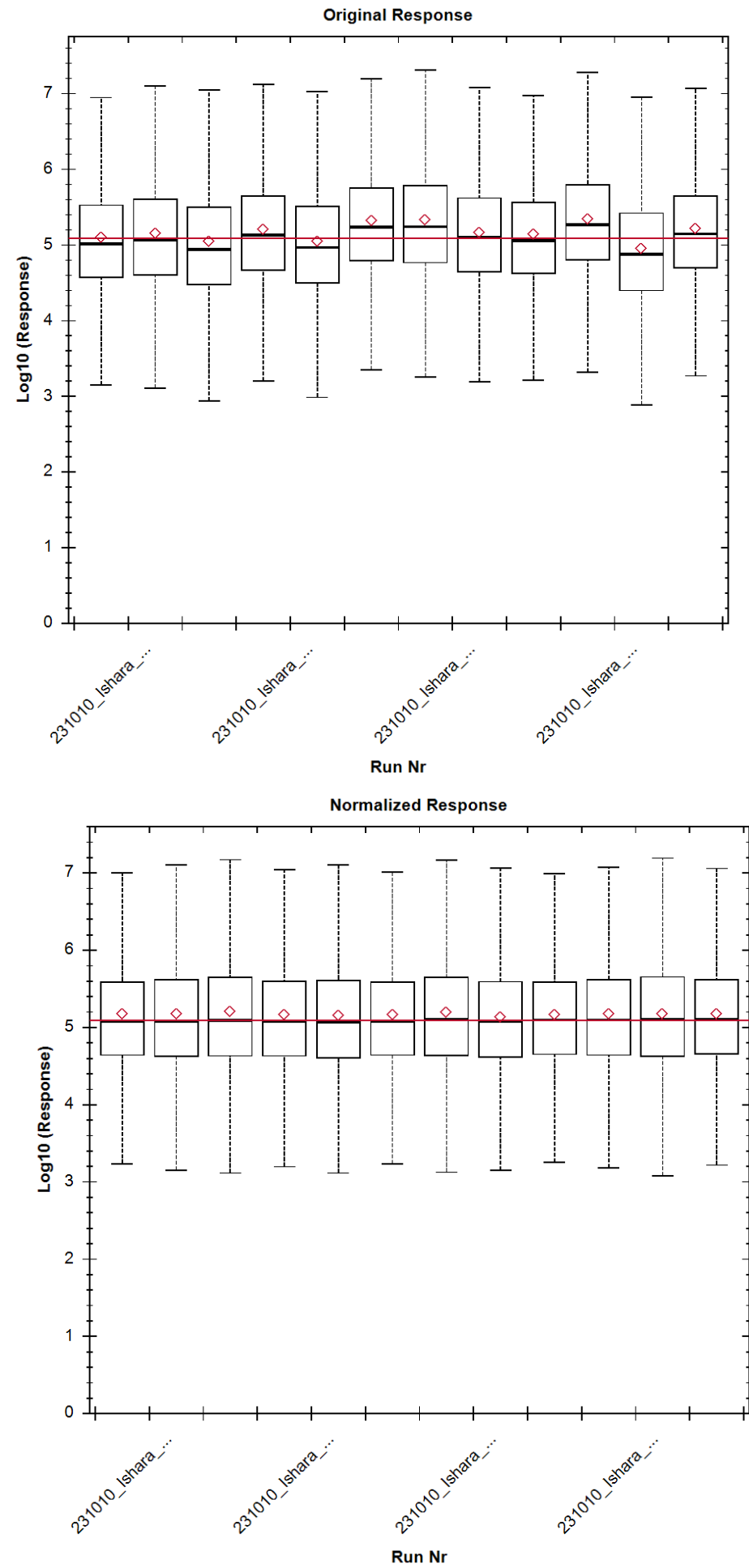

Supplement: Supplementary file 3 — Supplementary Figure 1 Pre‐ and post‐normalisation plots for the proteomic data. Box and whisker plots showing the data distribution for the 12 samples, before and after normalisation, using automatic cross‐run normalisation in Spectronaut software (v. 17.5.230413.55965). [file JEX2-4-e70085-s002.pdf]

Supplementary figure 2

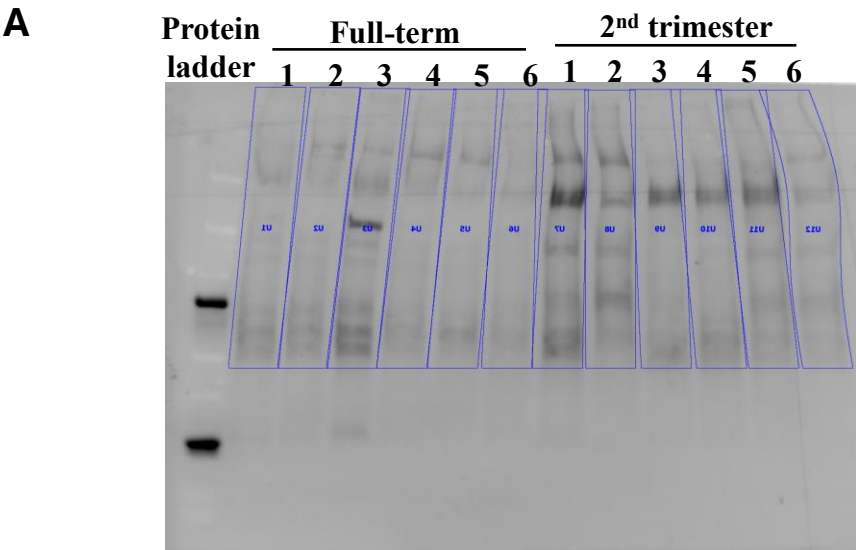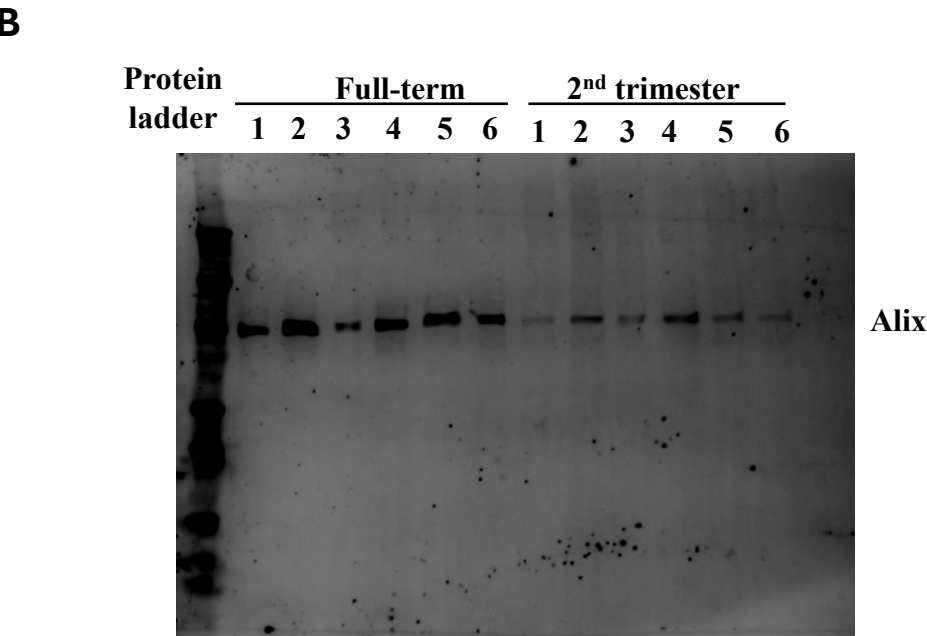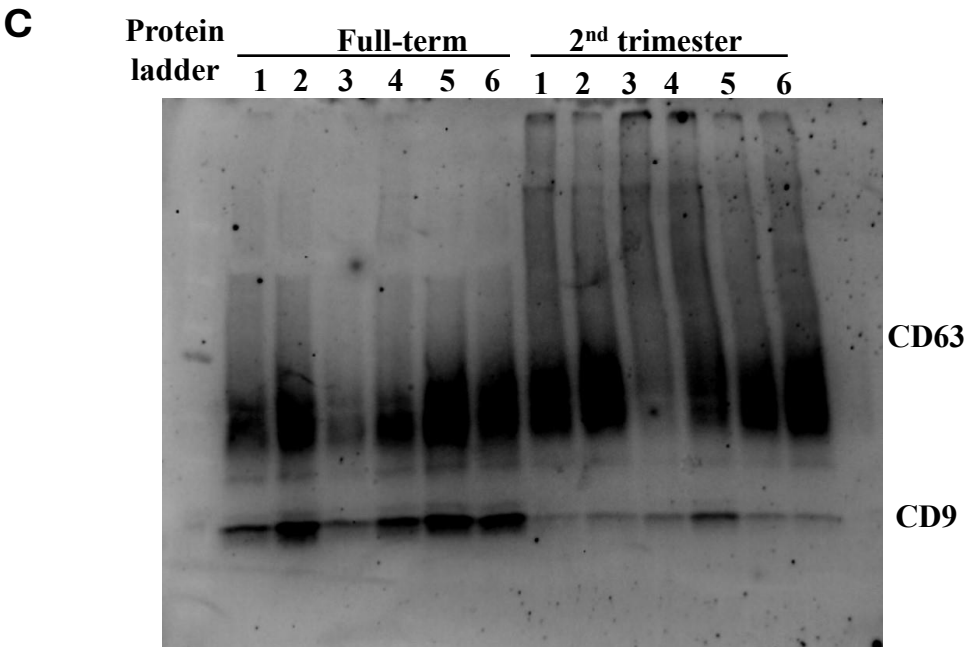

Supplement: Supplementary file 4 — Supplementary Figure 2 Sypro Ruby staining and densitometry of the complete Western blot. Western blot was stained with Sypro Ruby blot stain and analysed using Image Lab software (Bio‐Rad). (A) The protein ladder is on the first lane, followed by term samples and second‐trimester samples. Please note that this Western blot was cropped to separate the 2 sample groups and switched sides to obtain Figure 1A. (B) Complete Western blot with probing for Alix. (C) Complete Western blot with probing for CD63 and CD9. [file JEX2-4-e70085-s003.pdf]

Supplementary figure 3

A

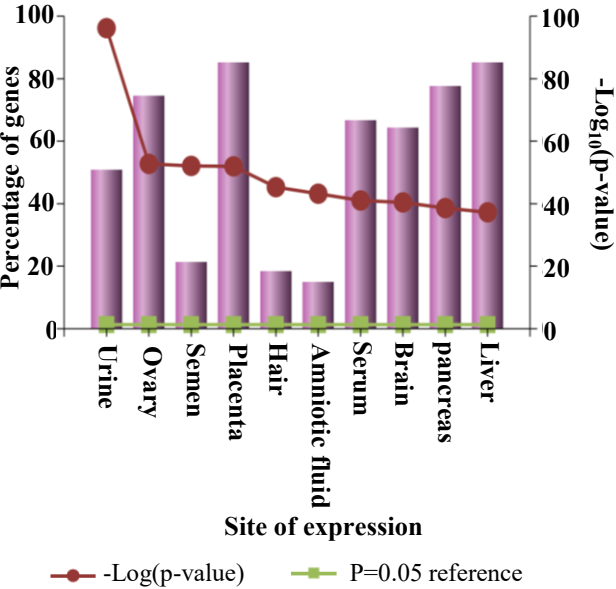

C

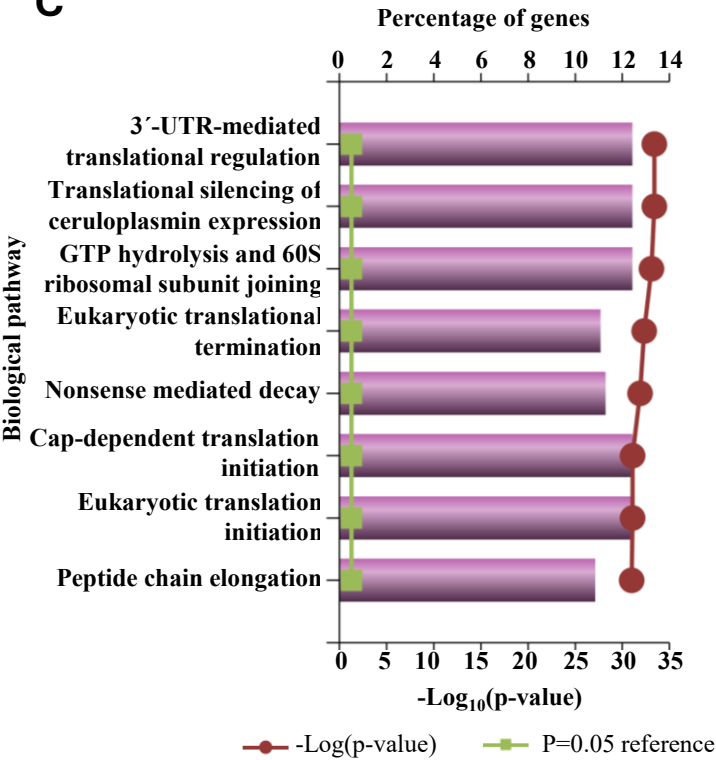

B

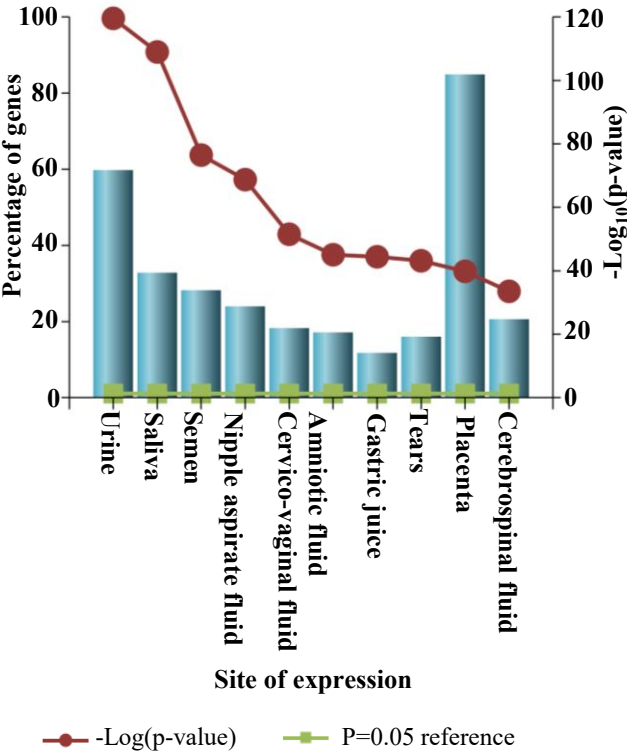

D

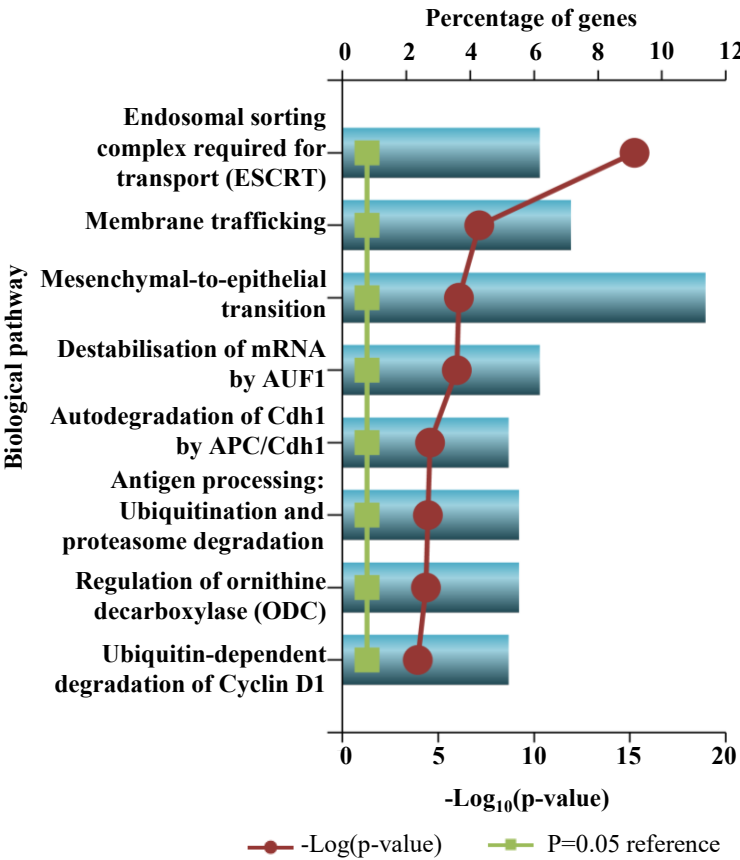

Supplement: Supplementary file 5 — Supplementary Figure 3 Protein enrichment analysis excluding the sample with a congenital anomaly. Enrichment analysis (Funrich) performed in Figure 4 was repeated for five pairs, excluding the second‐trimester sample with bilateral cleft lip and palate and a fetal sex‐matched control. Proteins uniquely expressed and significantly abundant in (A) second trimester and (B) term were matched with distinct site‐specific protein signatures. Nine out of the ten sites enriched in both gestations overlapped with their original analyses results presented in Figures 4A and 4B. A similar analysis was conducted to identify biological pathways enriched in (C) the second trimester and (D) term. Compared to their original analysis, there were no changes in the enriched biological pathways at both gestations in this 5‐pair analysis. [file JEX2-4-e70085-s005.pdf]
